# Editorial: Methods of engagement of dementia care users in research and practice development

**DOI:** 10.3389/frdem.2025.1692225

**Published:** 2025-09-23

**Authors:** W. George Kernohan, Suzanne Timmons, Anthea Innes, David Edvardsson

**Affiliations:** ^1^Ulster University, Belfast, United Kingdom; ^2^University College Cork, Cork, Ireland; ^3^Centre for Rural Health Science, University of the Highlands and Islands, Inverness, United Kingdom; ^4^Gilbrea Centre for Studies in Aging, Health, Aging and Society, McMaster University, Hamilton, ON, Canada; ^5^Swinburne University of Technology, Hawthorn, Australia

**Keywords:** personhood and dementia, user involvement in research, co-design benefits, evaluation of engagement, user involvement

## Introduction

Dementia is a growing global health challenge, affecting over 57 million people worldwide and placing increasing pressure on health and social care systems (World Health Organization (WHO), 2021). Despite the recognized value of involving people with dementia in coproduction and research, many researchers remain hesitant, often citing concerns about capacity, ethical complexity, or methodological limitations (Bethell et al., 2018). The imperative to involve people living with dementia and their care partners in research and practice development has gained increasing recognition in recent years. Participatory approaches, such as co-design and co-production, are now considered essential for creating interventions that are both meaningful and effective (Gove et al., 2018; Skivington et al., 2021).

The “*Methods of Engagement of Dementia Care Users in Research and Practice Development*” Research Topic in *Frontiers in Dementia* brings together articles that explore diverse strategies for involving people living with dementia and their supporters/caregivers in research and practice development. This editorial highlights the contributions of this Research Topic, aiming to explore and advance innovative methods for engaging people living with dementia and their families in the design, implementation, and evaluation of dementia care interventions. This body of work emphasizes participatory, co-design, and other collaborative approaches to research and practice development.

## Setting the scene: of gaps in research

We start with Bartels et al. who present a robust opinion piece identifying key methodological gaps in psychosocial dementia research. They critique the field's continued overreliance on RCTs, which frequently neglect context complexity, stakeholder involvement, and theory-driven mechanisms, leading to potential implementation failures and wasted resources. Their core call is for more stakeholder-informed, participatory, and mixed-method designs aligned with MRC phases. They also introduce the METHODEM initiative to systematically map and prioritize suitable methods. The paper urges methodological reform by blending conceptual clarity with suggested action. By incorporating diverse research designs and prioritizing meaningful stakeholder engagement, they and we propose a more holistic and effective approach for co-creating, evaluating and testing interventions that can be seamlessly translated into everyday practice.

## Valuing lived experience and personhood

Several other papers underscore the ethical imperative of fully recognizing people with dementia as persons with human rights. This includes adopting approaches like the *intentional stance* (O'Shea et al.) and valuing emotional, social, and identity-based outcomes of participation (Seidel et al.). This reframes engagement as an ethical, relational process rather than merely a technical exercise. Drawing on a single, powerful case study, O'Shea et al. illustrate how respectful, open-ended interaction can help temporarily bridge cognitive and communicative divides. Their integration of philosophical and methodological insights provides a valuable contribution to advancing inclusive dementia research practices and challenges the norm of passive participant roles.

Lived experience was not only acknowledged but integrated to improve the relevance of tools and research design (Donnelly et al.). Indeed, our Research Topic highlights the ethical dimensions of inclusion, such as informed consent (Diaz et al.), representation across dementia stages (Snowball et al.), and the need to avoid epistemic injustice (O'Shea et al.). This reflects a move toward flexible, person-centered research methods that uphold autonomy and dignity.

## On co-design and participatory methods

Co-creation of research instruments and dissemination strategies emerges as a practical and empowering method. Donnelly et al. show how co-design improved survey usability. By collaborating with a research advisory group comprising people with Lewy body dementia and their supporters/caregivers, the researchers co-designed a survey that was both accessible and relevant to the target population. Their pragmatic approach to involving people with dementia in research used a hybrid method that combined focus groups and interviews within a single event, addressing resource constraints while still capturing valuable feedback. This involvement led to tangible improvements in the survey's design, such as clearer attribute descriptions and more user-friendly presentation. De Wolf-Linder et al. illustrate co-production across all research phases, not just design or data Research Topic. They present a new model for engaging stakeholders in the dissemination of dementia research, promoting inclusivity and practical application of findings. Snowball et al.provide practical strategies for facilitating meaningful engagement, such as prioritizing accessibility and fostering an inclusive environment, underscoring the importance of integrating diverse voices to enrich the research process and outcomes.

## Evaluating engagement and impact

Several studies moved beyond participation to measure the *quality and effects* of engagement. Wong et al. used PEIRS-22 to track involvement quality, while Seidel et al. explored psychosocial outcomes of advisory group participation. Their participants reported enhanced self-perception of competence, feelings of joy and wellbeing, and increased social engagement. Notably, the study also acknowledges instances of sadness and insecurity, highlighting the complex emotional landscape of such involvement. Evaluation efforts indicate an increasing emphasis on accountability and learning in engagement practices.

## Enhancing communication

Effective engagement depends on reciprocal, accessible communication. Techniques like *Music Mirrors* (Edwards et al.) show the potential of integrating personalized audio-biographical cues into dementia care practices to enhance the quality of interactions. Their findings indicate that the use of Music Mirrors led to an improvement in the wellbeing of people with dementia, irrespective of the care environment.

It is clear that conversational strategies grounded in selfhood theory (O'Shea et al.) support meaningful interaction. Diaz et al. emphasize tailoring consent processes with lived-experience insight, especially in the context of new ethical challenges like AI. The authors argue that involving people with dementia and their supporters/caregivers in designing consent procedures can lead to more ethical and effective research practices, and that there is a need for more practical strategies for implementing inclusive consent processes and ensuring broader representation.

Engagement is also framed as a route to societal participation, not just research contribution. The Polish dementia campaign (Błaszkiewicz et al.) demonstrates that involvement fosters social health, belonging, and emotional wellbeing—reinforcing research as a vehicle for inclusion.

## Conclusion

Across the papers, a strong convergence emerges around **inclusive, ethical, and relational approaches** to involving people with dementia across the whole spectrum of research. Authors advocate for **moving beyond tokenism** toward co-created, evaluated, and socially embedded models of research. These studies push the field to prioritize **dignity, agency, and meaningful connection**, not only in methodology but in the broader purpose of dementia research.
